# A missense mutation in *zbtb17* blocks the earliest steps of T cell differentiation in zebrafish

**DOI:** 10.1038/srep44145

**Published:** 2017-03-07

**Authors:** Divine-Fondzenyuy Lawir, Norimasa Iwanami, Michael Schorpp, Thomas Boehm

**Affiliations:** 1 Department of Developmental Immunology, Max Planck Institute of Immunobiology and Epigenetics, Stuebeweg 51, D-79108 Freiburg, Germany

## Abstract

T cells are an evolutionarily conserved feature of the adaptive immune systems of vertebrates. Comparative studies using evolutionarily distant species hold great promise for unraveling the genetic landscape underlying this process. To this end, we used ENU mutagenesis to generate mutant zebrafish with specific aberrations in early T cell development. Here, we describe the identification of a recessive missense mutation in the transcriptional regulator zbtb17 (Q562K), which affects the ninth zinc finger module of the protein. Homozygous mutant fish exhibit an early block of intrathymic T cell development, as a result of impaired thymus colonization owing to reduced expression of the gene encoding the homing receptor ccr9a, and inefficient T cell differentiation owing to reduced expression of *socs1a*. Our results reveal the *zbtb17*-*socs1* axis as an evolutionarily conserved central regulatory module of early T cell development of vertebrates.

Forward genetic screens represent an unbiased experimental strategy that enables researchers to reconstruct the genetic landscape underlying a particular physiological process. Owing to its high fecundity and the low costs of animal maintenance, the zebrafish has emerged as an animal model for the genetic and functional analysis of various developmental processes in vertebrates. In our laboratory, we have carried out a large-scale forward genetic screen of ENU-mutagenized zebrafish to identify regulators of early T cell development (for instance, see ref. 1). Our screen was designed such that aberrations affecting haematopoietic progenitors can be identified up to the stage when developing thymocytes rearrange their TCRs. This was accomplished by subjecting zebrafish embryos derived from carriers of mutations to RNA *in situ* hybridization with a *rag1* probe; the expression of *rag1* marks the presence of immature T cells in the thymus^2^.

Here, we report on the identification and characterization of zebrafish carrying a missense mutation in the *zbtb17* gene. In mammals, *Zbtb17*, also known as *Miz*-*1*, has been shown to be expressed in all cells and to encode a transcription factor containing an N-terminal BTB domain and several C-terminal zinc fingers. Inactivation of *Zbtb17* in mice causes embryonic lethality^3^, compatible with the notion that it is involved in the regulation of the cell cycle^4^,^5^. Conditional ablation of *Zbtb17* in certain cell lineages of mice indicates its tissue-specific role in lymphopoiesis, specifically in regulating IL-7 signaling both in B cells^4^,^6^ and T cells^7^,^8^. With respect to B cell development, the lymphopoietic function of *Zbtb17* was attributed to its ability to repress the transcription of the gene encoding the Jak-inhibitor Socs1 and to activate *Bcl2* gene expression^6^,^7^, both effects synergizing to enhance the pro-lymphopoietic effects of IL-7 signaling. In addition, *Zbtb17* appears to affect T cell development independently of IL-7 signaling by regulating the surface expression of the pre-TCR^8^, suggesting a complex mode of action of the transcription factor. Of note, the mouse phenotypes reported so far are the consequence of null alleles of *Zbtb17*, complicating the derivation of genotype-phenotype correlations. Our zebrafish screen aimed at discovering genes required for T cell development indicated that a missense mutation in *zbtb17* is associated with failing T cell development. Here, we demonstrate an evolutionarily conserved function of *zbtb17* in T cell development of vertebrates.

## Results

### Identification of a missense mutation in the *zbtb17* gene

**Figure 1:**
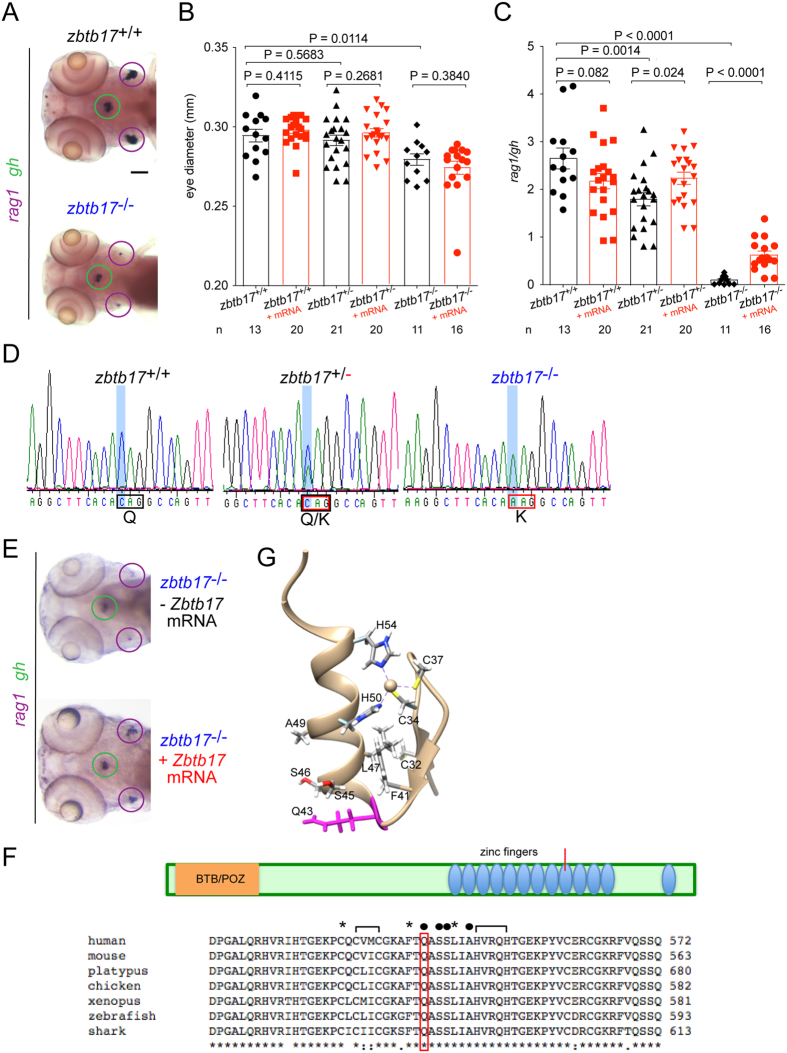
Impaired T cell development in fish homozygous for a missense mutation in *zbtb17*. (**A**) Representative microphotographs of wild-type (top panel) and homozygous mutant (bottom panel) zebrafish embryos hybridized with probes specific for *rag1* (purple circle) and *gh* (green circle) at 5 dpf. (**B**) Eye diameter (means ± s.e.m.) for the three different *zbtb17* genotypes (numbers of fish indicated) without (black bars) and with injection of mouse *Zbtb17* wild-type mRNA (mRNA) determined at 4 dpf. (**C**) Thymopoietic indices (means ± s.e.m.) for the three different *zbtb17* genotypes without (black bars) and with injection of mouse *Zbtb17* wild-type mRNA (mRNA) determined at 4 dpf. (**D**) Representative sequence traces covering part of exon 10 sequences of wild-type fish (left panel), heterozygotes (middle panel) and homozygous mutants (right panel). The C > A transversion occurs at nucleotide position 1684 of ENSDARG00000074548 (http://www.ensembl.org/Danio_rerio/Transcript/Summary?db=core;g=ENSDARG00000074548;r=23:24525127-24540083;t=ENSDART00000114840). (**E**) Representative microphotographs of *zbtb17* mutant embryos after injection of mouse *Zbtb17* mRNA hybridized with probes specific for *rag1* and *gh* at 96 hpf. The thymic rudiments (marked by expression of *rag1*) are encircled in purple, the hypophysis (marked by expression of *gh*) is encircled in green. (**F**) Schematic of the ZBTB17 proteins, indicating the position of known domains, including the 12 zinc finger modules. Below, an alignment of partial protein sequences of vertebrate ZBTB17 proteins around the critical region of the ninth zinc finger (marked in schematic) is shown. The presumptive zinc-coordinating (brackets), DNA-contacting (solid circles) and hydrophobic (asterisks) amino acid residues are marked. Source sequences: human, *H. sapiens*, Genbank accession number NP_001274532.1; mouse, *M. musculus*, Genbank accession number NP_033567.2; platypus, *Ornithorhynchus anatinus*, Genbank accession number XP_003429656.1; chicken, *G. gallus*, Genbank accession number NP_001263229.1; xenopus, *X. tropicalis*, Genbank accession number NP_001011141.2; zebrafish, *D. rerio*, Genbank accession number XP_003201251.1; shark, *C. milii*, Genbank accession number XP_007905152.1. (**G**) Structure of the ninth zinc finger of the mouse Zbtb17 protein (source code 2LVT); the mutated Q43 residue is indicated in purple. Scale bar, 100 μm.

The HK017 fish line harbors a recessive mutation that severely impairs T cell development, as evident from a much reduced hybridization signal in whole mount RNA *in situ* hybridization at 5 days after fertilization (dpf) with *rag1*, a marker of immature lymphoid cells rearranging their antigen receptor loci (Fig. 1A). However, the mutation has more widespread effects; for instance, as compared to their wild-type counterparts at 4 dpf, the mutant fish are slightly smaller, as exemplified by the reduced diameter of the eyes (Fig. 1B; black bars). Although the number of growth hormone (*gh*) gene-expressing cells in the hypophysis is also reduced in the mutants, the deleterious effect of the mutation on T cell differentiation is much stronger (Fig. 1A). At 4 dpf, the *rag1*/*gh* ratio (here designated thymopoietic index) amounts to only 9.33 ± 0.074% of the wild-type values (Fig. 1C; black bars). In *zbtb17*^+/−^ heterozygotes, the eye diameter is indistinguishable from wild-types; by contrast, we observed an approximately 30% reduction of the thymopoietic index supporting the notion that developing T cells are particularly vulnerable to reduced levels of *zbtb17* (Fig. 1B,C). Meiotic recombination mapping indicated that the mutation in the HK017 fish line is located on chromosome 23; the critical interval of about 0.11 cM (4 recombination events in 3600 meioses), corresponded to a region of approximately 310 kb, encompassing several genes, including *zbtb17*. It was found that a missense mutation in exon 10 of the *zbtb17* gene (ENSDARG00000074548) (Zv9; nucleotide 1684: C>A; ENSDART00000114840 [Fig. 1D]) segregated with the phenotype. As expected, 100/100 fish with a low thymopoietic index exhibited this mutation (P < 10^−32^, Fisher exact probability test). This allele is designated as *zbtb17*^t20463^. Next, we attempted to rescue failing T cell development in zebrafish mutants using the mouse wild-type RNA, to also test for an evolutionarily conserved function of *ZBTB17*. Provision of mouse *Zbtb17* mRNA partially rescued T cell development in the *zbtb17* mutant background and alleviated the haploinsufficiency state (Fig. 1C,E); under these conditions, no effect was seen for the eye phenotype (Fig. 1B), pointing to a differential requirement for *zbtb17* in different tissues.

Importantly, the inter-specific rescue of impaired T cell development suggests that the mouse *Zbtb17* gene functions during development of the evolutionarily distant zebrafish, compatible with the strong conservation of protein sequences of vertebrate ZBTB17 proteins. The missense mutation in the *zbtb17*^t20463^ allele occurs in the ninth of 13 zinc fingers of the ZBTB17 protein and affects an evolutionarily conserved glutamine residue (Fig. 1F) that is converted to lysine (Q562K), thus replacing an uncharged, polar side-chain with a positively charged one on the surface of the zinc finger module (Fig. 1G)^9^.

**Figure 2:**
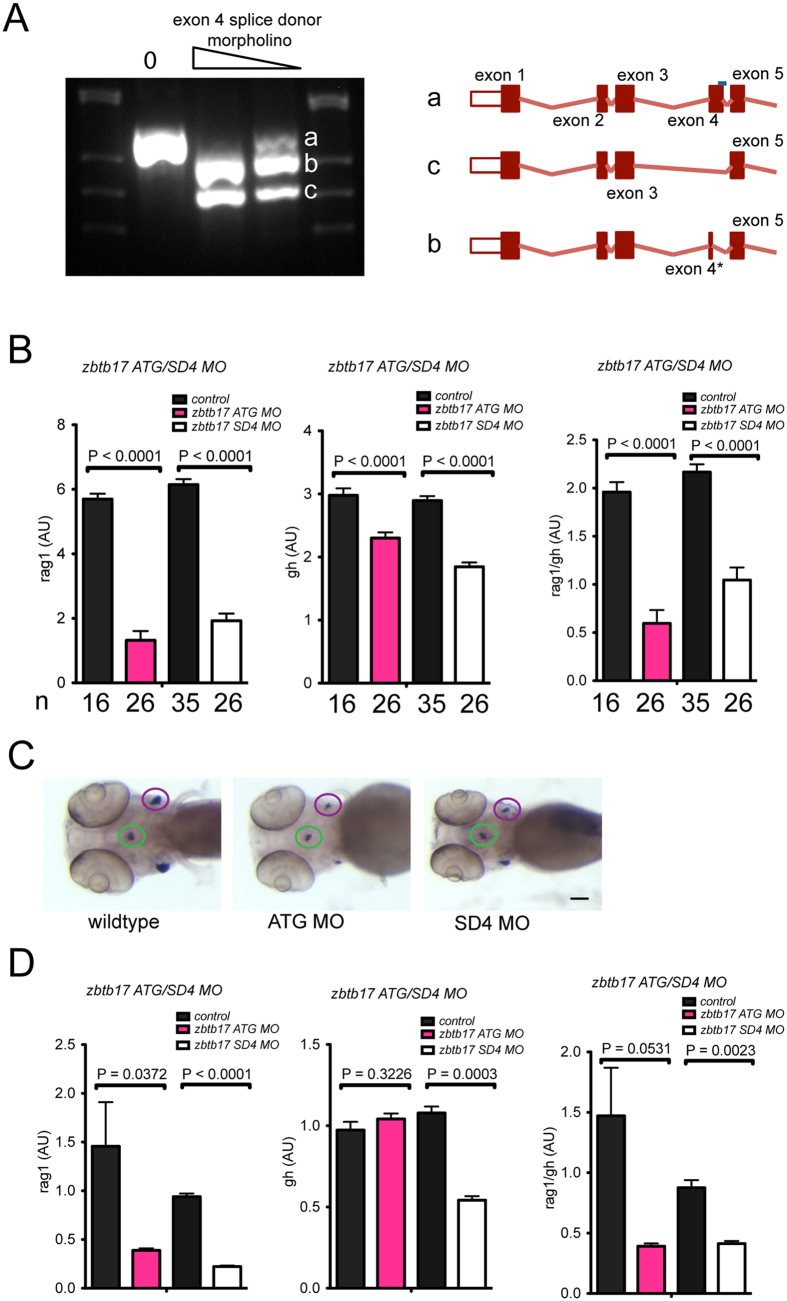
Characterization of *zbtb17* morphants. (**A**) Analysis of aberrant splicing patterns in exon 4 splice donor morphants. Representative pattern indicating wild-type (a) and two aberrant (b,c) cDNAs, whose structures are shown on the right. Isoform b originates from the use of a cryptic splice donor site in exon 4 (nucleotides 763–764 in transcript ENSDART000000114840.3); isoform b represents an exon-skipping event eliminating exon 4. (**B**) Thympoietic activity measured by RNA *in situ* hybridization in *zbtb17* morphants. The number of embryos in each cohort is indicated. (**C**) Representative microphotographs of *zbtb17* morphants (from (**B**)) after RNA *in situ* hybridization with *rag1* and *gh* probes (see Fig. 1). Scale bar, 100 μm. (**D**) Thymopoietic activity measured by qPCR in *zbtb17* morphants at 4 dpf. RNAs were isolated from pools of 20 embryos each.

Owing to our limited information on structure/function relationships of the zbtb17 protein, it is at present not possible to confidently predict the functional consequences resulting from the Q562K missense mutation, which might affect the DNA-binding and/or protein interaction properties of the ZBTB17 protein. To address the functional consequences of the *zbtb17* mutation further, we compared the phenotype of the *zbtb17*^Q562K^ mutant to those of *zbtb17* morphants. Two types of morphants were generated (Fig. 2). First, we used an oligonucleotide targeting a splice site in the *zbtb17* pre-mRNA (splice donor site of exon 4); in these SD-morphants, only nascent zygotic transcripts should be affected and maternally deposited (that is, already processed) *zbtb17* mRNAs spared and remain functional (Fig. 2A). Second, we employed an oligonucleotide targeted at the translation initiation site of *zbtb17* mRNAs, hence blocking both maternal and zygotic mRNAs (here designated ATG-morphants). Under both conditions, *rag1* expression levels were reduced (Fig. 2) as determined by RNA *in situ* hybridization (Fig. 2B,C) and qPCR (Fig. 2D), indicating that the morphants exhibited the desired phenotype. As expected, we observed that the expression levels of the *gh* gene are reduced in mutant and morphants; however, when we calculated the thymopoietic index based on gene expression levels (*rag1*/*gh*), we found the expected reduction, indicating the disproportionate effect of *zbtb17* deficiency on T cell differentiation (Fig. 2B,D; rightmost panels). This finding validates the determination of thymopoietic activity based on signals in RNA *in situ* hybridizations (Fig. 1A,B). Taken together, the results indicate that the zbtb17^Q562K^ mutant encoded by the *zbtb17*^t20463^ allele represents a variant with no (or reduced) activity.

### socs1a and socs3a are required for early T cell development

**Figure 3:**
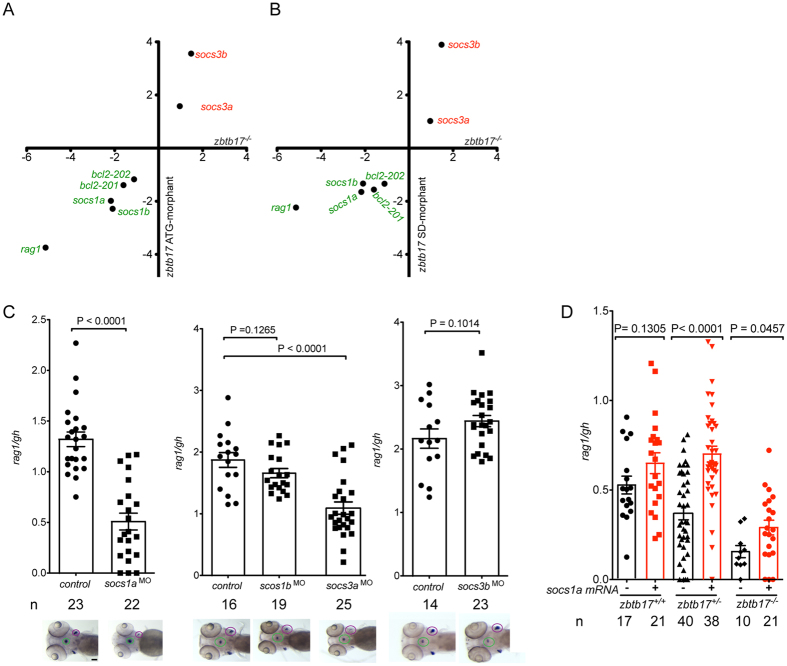
Functional consequences of the *zbtb17* missense mutation. (**A**) qPCR analysis of gene expression levels in homozygous *zbtb17* mutants at 5 dpf compared to gene expression levels for *zbtb17* ATG-morphants determined at 96 hpf. Expression levels were normalized to those of the *actb1* gene and are expressed as fold changes relative to wild-type values; mean values (n = 3 or 4) are shown (detailed results are presented in Supplementary Fig. 1). (**B**) qPCR analysis of gene expression levels in homozygous *zbtb17* mutants at 5 dpf compared to gene expression levels for *zbtb17* SD-morphants determined at 96 hpf. Expression levels were normalized to those of the *actb1* gene and are expressed as fold changes relative to wild-type values; mean values (n = 3 or 4) are shown (detailed results are presented in Supplementary Fig. 1). (**C**) Effect of *socs* gene knock-downs on thymopoietic activity expressed as *rag1*/*gh* ratios. Representative RNA *in situ* hybridization results (the thymic rudiments [marked by expression of *rag1*] are encircled in purple, the hypophysis [marked by expression of *gh*] is encircled in green) are shown underneath the relevant bar charts for which mean ± s.e.m. values are shown; each data-point represents one embryos (the total numbers of embryos for each column are indicated). Scale bar, 100 μm. (**D**) Rescue of the *zbtb17* mutant phenotype by provision of *socs1a* mRNA. The extent of T cell development was assessed by *rag1*/*gh* ratios after RNA *in situ* hybridization without (black bars) and with (red bars) provision of *in vitro* transcribed *socs1a* mRNA (the total number of embryos for each column are indicated).

In order to assess the molecular phenotype of *zbtb17* mutant and morphants in more detail, we determined the expression of several genes whose expression levels were previously shown to be dysregulated in mouse *Zbtbt17*-deficient lymphocytes^6^,^7^. We found that the expression levels of the two paralogous zebrafish *bcl2* genes were reduced (Fig. 3A,B). Variable effects were observed for the two paralogs of *socs1* and *socs3* genes, respectively: *socs1a* and *socs1b* expression levels were reduced about 2-fold; *socs3a* levels were essentially unchanged, whereas *socs3b* levels increased several-fold (Fig. 3A,B; see Supplementary Fig. 1 for detailed results). Importantly, changes in gene expression levels were concordant between mutant and morphants (Fig. 3A,B), further supporting the notion that the phenotypes of mutant and morphants are similar.

The above results suggest that the effects of the zbtb17^Q562K^ mutant on T cell development could be mediated, at least in part, by altered expression levels of the *socs*-like genes^10^,^11^,^12^,^13^. In order to examine this possibility further, we generated the respective morphants and determined their thymopoietic activities. Interestingly, T cell development is affected only in *socs1a*^13^ and *socs3a*, but not in *socs1b* and *socs3b* morphants (Fig. 3C), excluding a contribution of reduced *socs1b* levels to the phenotype of *zbtb17* deficiency. Moreover, since expression of *socs3a* is unchanged in *zbtb17* mutants, it appears that the function of *socs3a* is not connected to the phenotype of the zbtb17^Q562K^ mutant and thus falls outside the scope of the present study. These considerations focused our interest on *socs1a* as a possible downstream effector of *zbtb17* in regulating early T cell development.

Taken together, the results of the above experiments suggest that *zbtb17* is required for the activation of *socs1a* expression at an early stage of T cell development. If so, overexpression of *socs1a* should alleviate the detrimental effect of zbtb17^Q562K^ on T cell development. This proved to be the case. Provision of *socs1a* mRNA increased the thymopoietic index in zbtb17^Q562K^ heterozygous and homozygous fish (Fig. 3D), identifying *socs1a* as a functionally relevant mediator of *zbtb17* in early stages of T cell development. Collectively, these data allow us to infer a genetic network^11^, connecting *zbtb17* to *socs*-like genes: The transcription factor Zbtb17 directly or indirectly inhibits expression of *socs3b*, and activates *socs1a* and *socs1b* expression.

### Early haematopoietic development in *zbtb17* mutants

**Figure 4:**
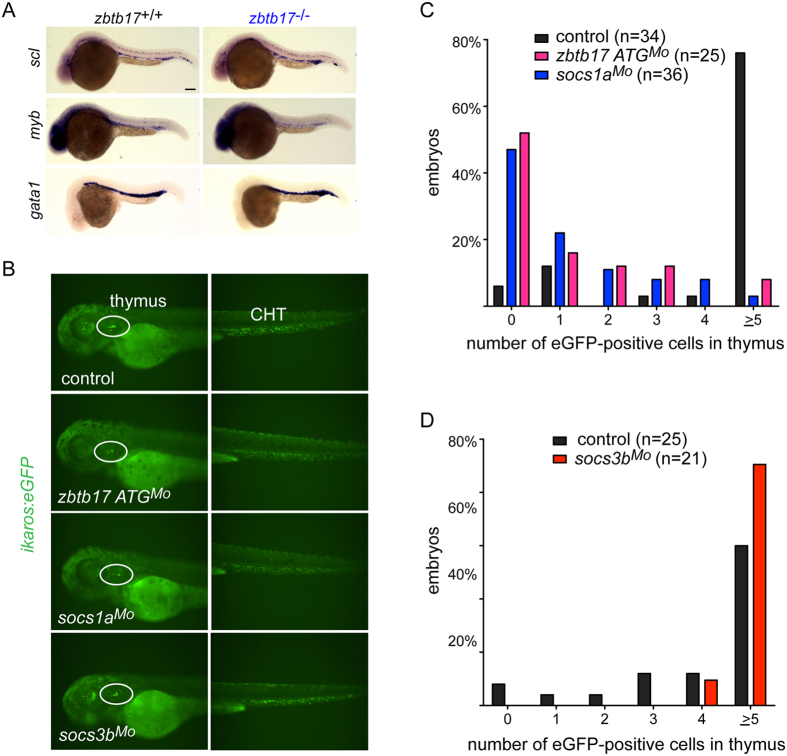
Unperturbed haematopoietic development in *zbtb17* mutants. (**A**) Microphotographs of wild-type and *zbtb17* mutant embryos after RNA *in situ* hybridization with the indicated gene-specific probes and at the indicated times of development (*scl*, 36 hpf; *myb*, 36 hpf; *gata1*, 24 hpf). Scale bar, 100 µm (**B**) Representative microphotographs of control and morphant embryos on the *ikaros:eGFP* transgenic background to visualize lymphoid progenitors in the thymus and the caudal haematopoietic tissue (CHT) at 63 hpf. (**C**,**D**) Thymus homing assay in control and morphant embryos. Summary statistics of cohorts of embryos (examples in (**B**)) for cell counts at 63 hpf.

An important question left unanswered by the above studies was whether the impaired development of T cells in *zbtb17* mutant embryos was due to pre- or intrathymic defects or both. To this end, we first examined the expression pattern of early haematopoietic markers in *zbtb17* mutants and found only small changes, most notable a slightly reduced expression of *c*-*myb* and somewhat increased expression of the erythroid marker *gata1* (Fig. 4A). Up-regulation of *gata1* expression is associated with reduced *socs1a* expression^13^, compatible with our finding that *zbtb17* appears to act upstream of *socs1a* (Fig. 3D); it is, however, possible that increased *socs3b* expression levels in the mutants have a synergistic effect with respect to upregulation of *gata1* expression, since *Socs3* is a positive regulator of erythroid differentiation in mammals^14^. Importantly, when we examined the presence of lymphoid lineage cells in the caudal haematopoietic tissue using an *ikaros:eGFP* transgene, no reduction of lymphoid progenitors was observed in *zbtb17* mutants/morphants; likewise, no reduction was seen in *socs1a* and *socs3b* morphants (Fig. 4B). Hence, we considered the possibility that thymus homing of lymphoid progenitors might be affected in *zbtb17* mutants/morphants and *socs1a* morphants.

### Impaired thymus homing of T cell development in *zbtb17* mutants

In order to examine a possible defect of T cell progenitor homing, we created *zbtb17, socs1a* and *socs3b* morphants in the *ikaros:eGFP* transgenic background, enabling precise enumeration of homing progenitors^15^. *zbtb17* and *socs1a* morphants exhibited a strong reduction of migration; when assayed at 63 hpf, the thymi of about 80% of wild-type embryos contained 5 or more lymphoid progenitors, whereas the same was true for less than 10% of the two morphants (P < 0.001; X^2^-test, two-tailed; Fig. 4C). By contrast, it appeared that thymus homing was not impaired, possibly even stimulated, in *socs3b* morphants (Fig. 4D). Collectively, these results indicate that the *zbtb17*-*socs1a* axis contributes to the control of thymus homing in zebrafish.

### Impaired intrathymic T cell development in *zbtb17* mutants

**Figure 5:**
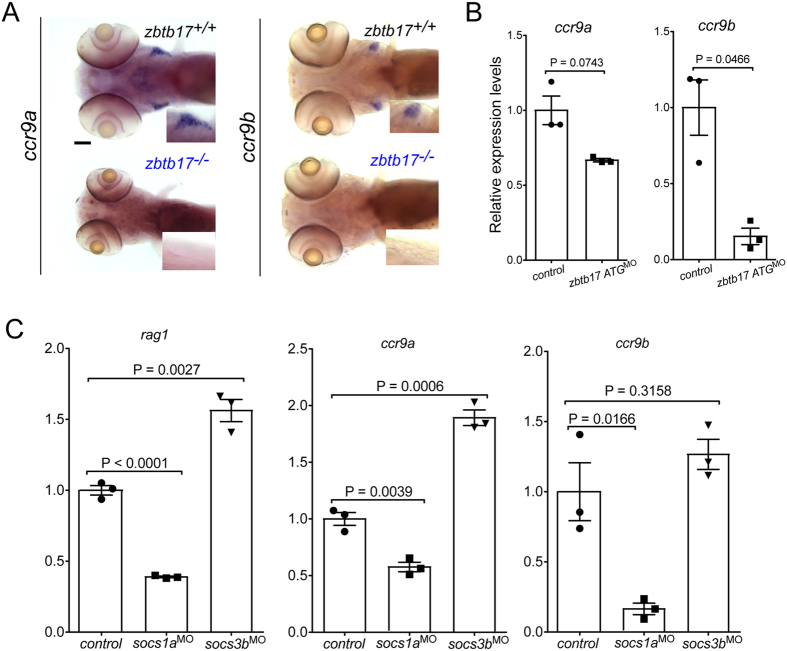
Chemokine receptor expression. (**A**) Representative microphotographs of wild-type and *zbtb17* mutant embryos after RNA *in situ* hybridization with *ccr9a* and *ccr9b* gene-specific probes at 5 dpf. Insets represent 2× magnifications of the thymic regions. No signal is detectable in *zbtb17* mutants. Scale bar, 100 μm. (**B**) Gene expression levels for *ccr9a* and *ccr9b* in *zbtb17* ATG-morphants as assessed by qPCR. (**C**) Gene expression levels for *rag1, ccr9a* and *ccr9b* in *socs1a* and *socs3b* morphants as assessed by qPCR.

Our previous work has indicated that *ccl25a* is the key effector of thymus homing in zebrafish, with *cxcl12a* making a minor contribution^15^. In order to gain insight into the mechanism of impaired homing, we determined the expression patterns of *ccr9a* and *ccr9b*, encoding the receptors for the ccl25a and ccl25b chemokines. The RNA *in situ* hybridization results for wild-type fish shown in Fig. 5A indicate that, whereas the expression of *ccr9b* is entirely confined to cells within the thymus, the expression of *ccr9a* is also present in cells in the vicinity of the thymic rudiment, as expected for a gene encoding a homing receptor. Accordingly, *ccl25a* is expressed in the vicinity of the thymus, whereas its paralog *ccl25b* is expressed only intrathymically and is not involved in the regulation of thymus homing^15^. In *zbtb17* mutants, expression of *ccr9a* and *ccr9b* genes was not detectable by RNA *in situ* hybridization (Fig. 5A). Hence, we examined the expression levels of *ccr9a* and *ccr9b* genes in ATG-morphants (where the titration of the anti-sense oligonucleotide allows us to generate a milder phenotype than that seen in the *zbtb17* mutants) (Fig. 5B). This result suggested that the reduced expression of the homing receptor gene *ccr9a* results in significantly reduced colonization of the thymus, contributing to failure of subsequent intrathymic differentiation. The latter effect is indicated by the fact that few if any thymocytes express *ccr9b, tcrb* or *tcrd*, supporting the notion that intrathymic T cell development stalls at a very early stage of differentiation (Supplementary Fig. 2A). To assess possible contributions of defective stromal components to this phenotype, we searched for signs of abnormal development of non-haematopoietic components of the pharyngeal region. However, no defects were noted using *gcm2* (the zebrafish homolog of the mouse *Gcm2* gene as a marker for zebrafish pharyngeal ectoderm), *foxn1* (the zebrafish homolog of the mouse *Foxn1* gene that is required for differentiation of thymic epithelial cells as a marker of endodermal derivatives), *dlx2* (as a marker for neural crest development), and alcian blue staining (as a reporter of cartilage formation) (Supplementary Fig. 2B).

Given that *zbtb17* appears to act upstream of *socs1a* and *socs3b* genes, we predicted that expression levels of *ccr9a* would also be affected in *socs1a* and *socs3b* morphants. Indeed, reduced expression of *ccr9a* and *ccr9b* was observed in *socs1a* morphants; like in *zbtb17* morphants, expression of *ccr9a* was less affected than *ccr9b* (Fig. 5C). This finding supports the notion that the *zbtb17*-socs1a axis affects T cell development prior to colonization of the thymus by T cell progenitors. Interestingly, increased expression of *ccr9a* was observed in *socs3b* morphants (Fig. 5C), providing an explanation for the greater number of thymocytes observed in *socs3b* morphants (Fig. 4D). Thus, *zbtb17* appears to regulate *ccr9a* expression by providing positive and negative influences (Supplementary Fig. 2C).

## Discussion

In zebrafish, T cell development in the thymus begins as early as 3 days after fertilization. This particular aspect of zebrafish embryogenesis has enabled the use of unbiased forward genetic screens for the identification of previously unknown regulators of early T cell development (for a recent review, see ref. 16). During our studies aimed at examining the genetic architecture of vertebrate lymphopoiesis, we identified the first mutant allele of zebrafish *zbtb17*. Its unique functional characteristics shed new light on early T cell development in zebrafish and reveal novel aspects of the striking degree of evolutionary conservation of early T cell development among distantly related vertebrates.

The similar phenotypes of *zbtb17* morphants and *zbtb17*^t20463^ mutants with respect to the extent of T cell development in the thymus, and the concordant changes in the expression levels of each of the two paralogs of *bcl2, socs1* and *socs3* genes suggest that the Q562K variant of zbtb17 is an amorphic protein; at present, however, we cannot exclude the possibility that it represents a hypomorphic version of the transcription factor. Previous functional analyses of *Zbtb17* have relied on the removal of whole protein domains during conditional mutagenesis in mice^4^,^6^,^7^,^8^, rendering the Zbtb17 protein non-functional. The present study represents, to the best of our knowledge, the first description of the functional relevance of a single amino acid in the Zbtb17 protein, highlighting the functional relevance of the Q562 residue located in the ninth zinc finger of the protein.

Our studies suggest that the zbtb17^Q562K^ variant fails, directly or indirectly, to properly regulate (among other genes) the transcription of the *socs1a* gene, which encodes a major regulator of different steps of T cell development. However, our studies give no indication as to the mechanism(s) by which *zbtb17* regulates *socs1a* gene expression. We note that all DNA target sites for mouse Zbtb17 described so far^7^ are considerably shorter than the (13 × 3=) 39 base pairs expected if each of the 13 zinc fingers of Zbtb17 contributed to the recognition of a triplet of base pairs according to the commonly accepted DNA recognition mode of zinc finger transcription factors^9^. Hence, the possibility exists that some (including the mutated ninth zinc finger identified here) instead contribute to protein binding, another well-known function of zinc finger-like protein modules^17^. Irrespective of the underlying mechanism by which the *zbtb17*^Q562K^ variant affects target gene transcription, our results clearly identify *socs1a* deficiency as a contributor to impaired T cell development and thus may explain, at least in part, the phenotype of fish homozygous for the *zbtb17*^t20463^ allele. Our detailed expression analyses indicate that the defect in *zbtb17* mutants affects the earliest stages of T cell development, a combination of impaired homing to the rudiment and impaired progression through the earliest stages of T cell development. However, these aberrations do not yield an absolute block, as some cells progress to the stage when they activate the *rag1* gene. Our results therefore raise the possibility that mouse Socs1 regulates *Ccr9* gene expression (and hence might affect thymus homing), in addition to its function during double-negative stages of T cell development, culminating in a block at the transition of DN3/4 cells to DP thymocytes^18^, a maturation defect compatible with the phenotype we observe in mutant zebrafish.

Notably, because *zbtb17* mutants die between days 12 and 14 after fertilization, it was not possible to examine potential effects on B cell development, which in zebrafish begins at the age of about 3 weeks^19^.

In conclusion, our findings suggest a role of *ZBTB17* in the regulation of thymus homing and firmly root *zbtb17* and *socs1a* in the genetic landscape regulating early steps of the T cell development of zebrafish. Our data thus reveal that the *zbtb17*-*socs1* axis is an evolutionarily conserved central and pleiotropic regulatory module of T cell development of vertebrates.

## Methods

### Animals

The zebrafish (*D. rerio*) strains wildtype-in-Kalkutta (WIK), AB, and Tübingen (TÜ) are maintained in the animal facility of the Max Planck Institute of Immunobiology and Epigenetics. The HK017 line (*zbtb17*^t20464^) originates from the Tübingen 2000 screen. The *ikaros:eGFP* transgenic lines has been described^15^. All animal experiments were performed in accordance with relevant guidelines and regulations, approved by the review committee of the Max Planck Institute of Immunobiology and Epigenetics and the Regierungspräsidium Freiburg, Germany (license Az 35-9185.81/G-12/85).

### Linkage analysis and gene identification

The genomic localization of zebrafish mutations was determined using the Tübingen marker set for genome scans (version 4) on F_2_-Tübingen × WIK crosses of the mutant carriers. Primer sequences are available from zfin.org. The mutation in HK017 was mapped to zebrafish chromosome 23. For fine-scale mapping, new markers were generated as follows. Marker HK017_129 (situated at 24.37 Mbp; 3/3600 recombinants): HK017-129L (GTCACATCAGAAACCGGAATAC), HK017-129R (TCTCATCAGGGACACGCAATC). Marker HK017_116 (24.54 Mbp; 0/3600 recombinants): HK017-116L (CAGCTTTGTTCAGGTGGGAGT), HK017-116R (CTTGGCTCGAACCAATCAGCT). Marker HK017_121 (24.68 Mbp; 1/3600 recombinants): HK017-121L (GCCAGTACAGTGTCATCAACC), HK017-121R (GAGCCCTGCTTCATCTGATGA). Marker HK017_102 (24.83 Mbp; 2 recombinants in 3600 meioses): HK017_102L (GAGCTGCAGAGATCTTATCTGTC), HK017_102R (AGCTGTATAGAAGTGTCCTGA). Sequence coordinates are for genome assembly Zv9 available at http://www.ensembl.org/Danio_rerio/Info/Index?db=core. Thus, the critical interval spanned 0.11 cM and 0.31 Mbp. The *zbtb17* gene was identified in the critical interval and its coding exons (including flanking regions) were sequenced after PCR amplification from genomic DNA of phenotypically wild-type (that is, a mixture of wild-types and heterozygous fish) and mutant embryos, which were identified by prior RNA *in situ* hybridization with the *rag1* probe. Primer sequences used for these analyses are available upon request.

### Mutant characterization

Prior to detailed phenotypic analysis, fish were out-crossed with wild-type fish for several generations to eliminate the potential influence of any background mutations. Using molecular probes, all mutants were subsequently analyzed by RNA *in situ* hybridization at various time points during the first 5 days of embryonic development to identify any potential abnormalities of haematopoietic cells, development of pharyngeal endoderm and ectoderm, and of structures derived from the neural crest. Differentiation of haematopoietic cells in the intermediate cell mass, a site of embryonic blood formation, was assessed by hybridization with probes specific for *tal1*/*scl* (a gene that specifies haematopoietic and vascular progenitor cells)^20^, *gata1a* (a gene required for red blood cell development)^21^, *lcp1*/*l*-*plastin*, (a marker of the myeloid lineage)^22^, and *ikzf1*/*ikaros* (a putative marker of lymphoid progenitors in zebrafish)^23^. The arrival and early differentiation of T cell progenitors in the thymic rudiment were assessed by hybridization with probes specific for *ikzf1*/*ikaros, ccr9a*/*ccr9b* (the zebrafish homologs of the mammalian chemokine receptor 9, a marker of early T cells in the mouse)^24^,^25^, *rag1* (a marker of immature lymphoid cells rearranging their antigen receptor loci)^26^,^27^,^28^, and TCR (*tcrb* and *tcrd*) as markers of αβ and γδ T cells, respectively^29^. Development of the pharyngeal arches was analyzed using *gcm2*, the zebrafish homolog of the mouse *Gcm2* gene as a marker for zebrafish pharyngeal ectoderm^30^, and *foxn1*^31^, the zebrafish homolog of the mouse *Foxn1* gene that is required for differentiation of thymic epithelial cells^32^ and expressed in endodermal derivatives. Neural crest development was assessed by *dlx2* expression^33^ and cartilage formation by alcian blue staining. Homozygous mutant fish only survive until 14 dpf, precluding an analysis of B cell development that begins at a later stage^34^.

### Morphants

Morphants were generated by injection of anti-sense morpholino oligonucleotides (Gene Tools, Philomath, OR) to block translation of both maternal and zygotic mRNAs, or block splicing of zygotic pre-mRNAs. Stock solutions were diluted as required and the final concentration in the injection buffer indicated; approximately 1–2 nl of solution were injected into fertilized eggs as described^29^. The sequences for gene-specific morpholino antisense oligonucleotides are as follows: *zbtb17*-ATG, ATGGAAAATCCATAACTGACTGTGT [targeting the initiation codon] (0.1 mM); *zbtb17*-SD4, TACAAGACAGAATTTACCTTCTGCG [targeting the splice donor site of exon 4] (0.1 mM); *socs1a*, TGCGCCACCATCCTACAGGAAAGAC (0.1 mM)^35^; *socs1b*, CCATTCTGAACTGAAGCCCACTCAT (0.2 mM); *socs3a*, TGTGGGTTATCATGGCGATACACAC (0.1 mM); *socs3b*, TGTCAAGCCTACTATGCGTTACCAT (0.1 mM)^36^.

### Preparation of mRNAs for phenotypic rescue

Mouse *Zbtb17* mRNA (Genbank accession number BC145312; Image clone 9053867) was generated as follows: The plasmid was linearized with *Spe*I and sense-RNA transcribed from the T7 promoter using the mMESSAGE mMACHINE kit (Ambion). Capped mRNA was dissolved at 0.1 mg/ml. Zebrafish *socs1a* mRNA (Genbank accession number BC077158; Image clone IRBOp991D0542D) was generated as follows: The plasmid was linearized with *Xho*I and sense-RNA transcribed from the Sp6 promoter using the mMESSAGE mMECHINE Kit. Capped mRNA was dissolved at 0.2 mg/ml.

For rescue, 1–2 nl of the RNA solution was injected into 1-cell embryos derived from an incross of HK017 carriers and analysis carried out by *gh* and *rag1* RNA *in situ* hybridization 4 days after injection.

### RNA *in situ* hybridization

Procedures for RNA *in situ* hybridization and probes were described previously^29^.

### Thymopoietic index

Thymic *rag1* gene expression is a marker of ongoing assembly of T cell receptor genes. Hence, the intensity of the RNA *in situ* signal correlates with the number of differentiating T cells, which we consider to be a measure of T cell development. In order to provide an internal control (technical, with respect to the hybridization process, and, biological, with respect to the tissue specificity of the observed genetic effects), we employed a probe specific for the growth hormone (*gh*) gene, which marks a subset of cells in the hypophysis. Determination of *rag1*/*gh* ratios was carried out as follows: After RNA *in situ* hybridization with *rag1* and *gh* probes, ventral images of 4–5 dpf zebrafish larvae were taken on an MZFLIII (Leica) microscope using a digital camera DFC300FX (Leica), essentially generating a two-dimensional projection of the three-dimensional structure. The areas of *rag1* and *gh* signals were measured using ImageJ (NIH), and the ratio of average of the *rag1*-positive area vs *gh*-positive area was calculated as a measure of thymopoietic activity. After photographic documentation of the RNA *in situ* hybridization signal, larvae were processed for genomic DNA extraction for subsequent genotyping, where required.

### RNA extraction and cDNA synthesis

Total RNA was extracted using TRI Reagent (Sigma) following the manufacturer’s instructions. After treatment with DNaseI (Promega), RNA extraction using TRI Reagent was repeated. Superscript II Reverse Transcriptase (Invitrogen) and oligo(dT) were used for cDNA synthesis from total RNA.

### quantitative PCR

qPCR was carried out as described^37^ using SYBR Premix Ex Taq (Takara) and 7500 fast real-time PCR system (Applied Biosystems). The transcript identifier (ENSEMBL) is given in brackets; the following primers were used: *bcl2*-*1* (ENSDART00000128843), bcl2-202F1: AGTGGAGGAATCCTCTCC, bcl2-202R1: GAGCCTCAAATGAGGGTC; *bcl2*-*2* (ENSDART00000130300), bcl2-2/2-201F2: AGAGTTTATATCAGTCGGAC, bcl2-2/2-201:R2 ATTCTGCCCCAGTTCACG; *socs1a* (ENSDART00000055537), socs1a-001F2: TACCGTGGCTTTCCAGAC, socs1a-001R1 GGAAAGTTCCCACTGACTC; *socs1b* (ENSDART00000123246), socs1b-001F2: TGGATGTGGACGAAGCTC, socs1b-001R2: CGATAGCTGAGCGTGAAG; *socs3a* (ENSDART00000033716), socs3a-001F2: GGATCCCTCGAAGTTCCT, socs3a-001R2: TTGCTGGACACATCCGTG; *socs3b* (ENSDART00000037904), socs3b-001F2: CATCCAGTGCGATTCCTC, socs3b-001R2 CCTTCTCCAGCATTGGAG. For qPCR of *gh, ccr9a* and *ccr9b* transripts, Bio-Rad assays were used: qDreCID0011909, qDreCED0009932, and qDreCID0016262 respectively). Primer sequences for zebrafish *rag1* and *actb1* genes were taken from Lam *et al*.^38^. RNAs for qPCR analysis were extracted from whole embryos.

### Statistical analysis

*t*-tests (two-tailed) were used to determine the significance levels of the differences between the means of two independent samples, considering equal or unequal variances as determined by the *F*-test. For multiple tests, the Bonferroni correction was applied.

## Additional Information

**How to cite this article**: Lawir, D.-F. *et al*. A missense mutation in *zbtb17* blocks the earliest steps of T cell differentiation in zebrafish. *Sci. Rep.* **7**, 44145; doi: 10.1038/srep44145 (2017).

**Publisher's note:** Springer Nature remains neutral with regard to jurisdictional claims in published maps and institutional affiliations.

## Supplementary Material

Supplementary Information
