# Supplementary material for: A missense mutation in zbtb17 blocks the earliest steps of T cell differentiation in zebrafish
Source: Sci Rep. 2017 Mar 7;7:44145. doi: 10.1038/srep44145 (PMC5339814; doi:10.1038/srep44145)
Supplement: Supplementary Information [file srep44145-s1.pdf]

Supplementary information for

A missense mutation in *zbtb17* blocks the earliest steps of T cell differentiation in zebrafish

Divine-Fondzenyuy Lawir, Norimasa Iwanami, Michael Schorpp, Thomas Boehm

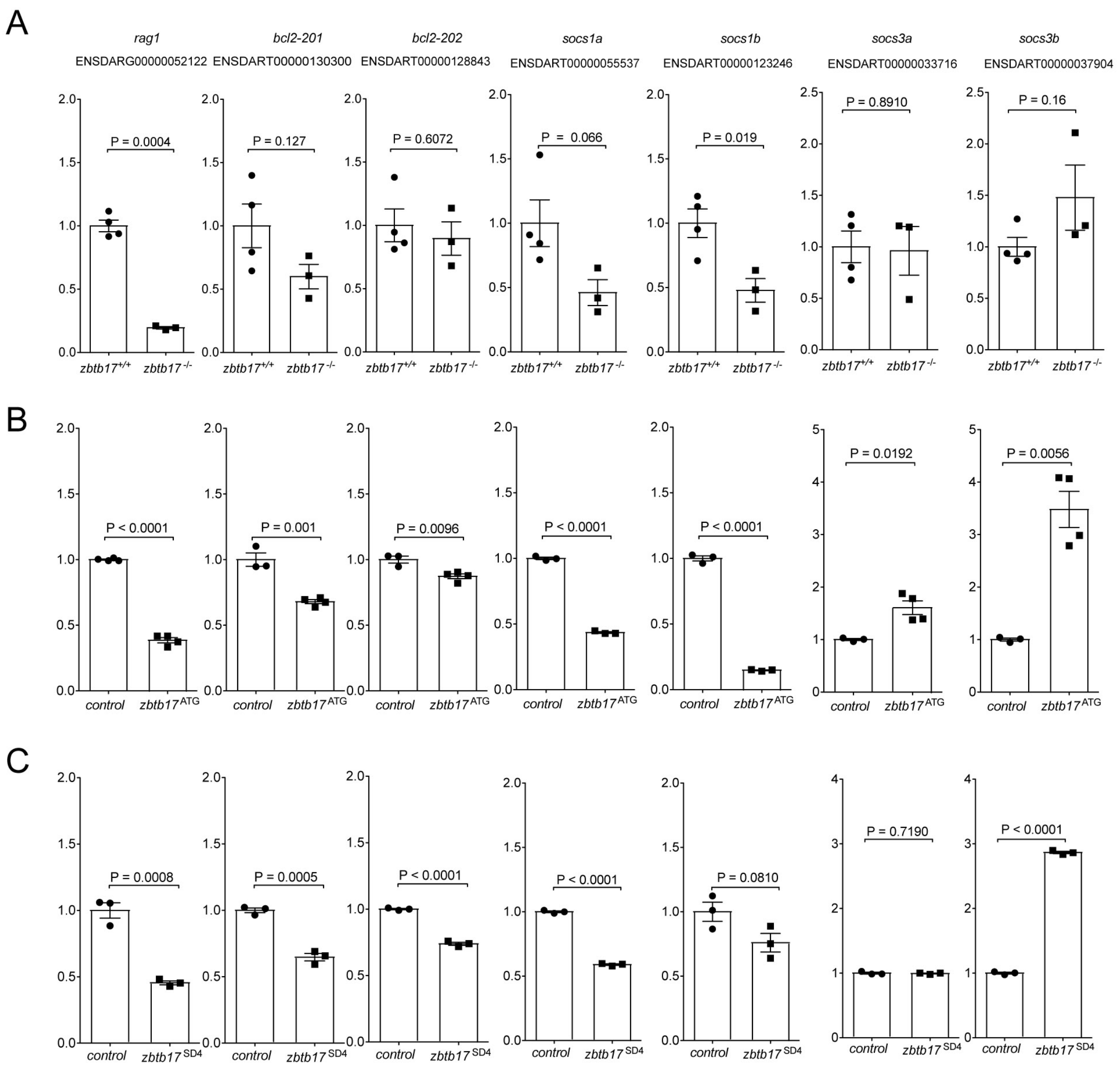

Supplementary Figure 1. Results of expression analysis in *zbtb17* mutants and *zbtb17* morphants. (**A**) qPCR analysis for *zbtb17* mutants of different genes at 4 dpf. The gene names and the respective gene identification numbers are given. Data are normalized to control samples. (**B**) qPCR analysis for *zbtb17* ATG morphants. (**C**) qPCR analysis for *zbtb17* SD4 morphants

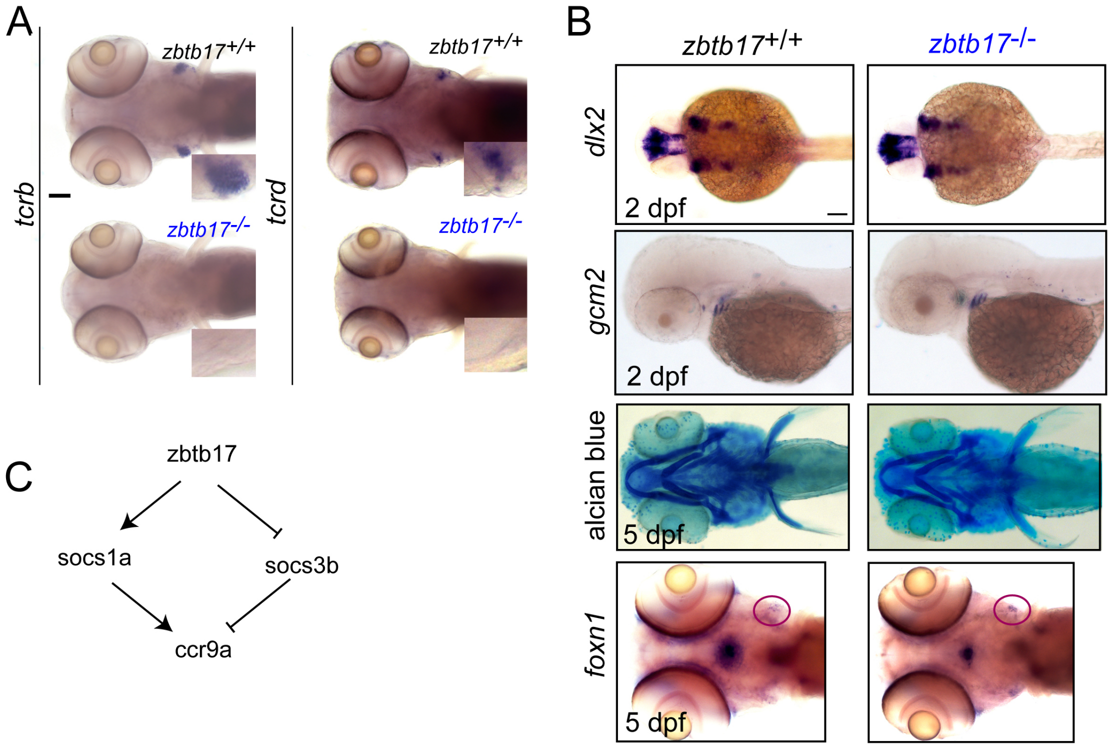

Supplementary Figure 2. Characterization of *zbtb17* mutants. (**A**) Representative microphotographs of wild-type and *zbtb17* mutant embryos after RNA *in situ* hybridization with *tcrb* and *tcrd* gene-specific probes at 5 dpf. Insets represent magnifications of the thymic regions. No signal is detectable in *zbtb17* mutants. (**B**) Microphotographs of wild-type and *zbtb17* mutant embryos after RNA *in situ* hybridization with the indicated gene-specific probes and at the indicated times of development. Alcian blue staining was used to visualize craniofacial cartilaginous structures. (**C**) Schematic of the network regulating *ccr9a* expression.
Scale bars, 100 µm.
